# Etanercept for steroid-refractory acute graft-versus-host disease: A single center experience

**DOI:** 10.1371/journal.pone.0187184

**Published:** 2017-10-26

**Authors:** Cornelis N. De Jong, Lotte Saes, Clara P. W. Klerk, Marjolein Van der Klift, Jan J. Cornelissen, Annoek E. C. Broers

**Affiliations:** 1 Department of Hematology, Erasmus Medical Center, Rotterdam, the Netherlands; 2 Department of Hematology and Oncology, Onze Lieve Vrouwe Gasthuis, Amsterdam, the Netherlands; 3 Department of Hematology, Amphia Hospital, Breda, the Netherlands; University of Kentucky, UNITED STATES

## Abstract

**Background:**

Acute graft-versus-host disease (aGVHD) is an important complication of allogeneic stem cell transplantation (alloSCT). High dose glucocorticosteroids, are currently recommended as first-line treatment for grade II-IV aGVHD resulting in overall complete responses (CR) in 40%-50% of patients. No standard second-line regimen has been established. Different options have been reported, including anti-TNFα antibodies.

**Methods:**

We retrospectively reviewed the outcome of 15 patients with steroid-refractory (SR) aGVHD treated with etanercept at our institution. Patients were transplanted for a hematological malignancy and received either a myeloablative or a non-myeloablative conditioning regimen. Prophylaxis of GVHD consisted of cyclosporin A and mycophenolic acid.

**Results:**

Acute GVHD was diagnosed at a median of 61 days post-transplantation. All patients had grade III aGVHD of the gut. Second-line treatment with etanercept was started at a median of 13 days after initiation of first-line therapy. Overall response rate was 53%, with CR in 3 patients and PR in 5 patients. Median overall survival after initiation of treatment with etanercept was 66 days (range 5–267) for the entire group. Median overall survival was 99 days (range 47–267 days) for responders and 17 days (range 5–66 days) for non-responders (p<0.01). Nevertheless, all patients died. Causes of death were progressive GVHD in 7 patients (47%), infection in 6 patients (40%), cardiac death in 1 patient (6.7%) and relapse in 1 patient (6,7%).

**Conclusion:**

Second-line treatment with etanercept does induce responses in SR-aGVHD of the gut but appears to be associated with poor long-term survival even in responding patients.

## Introduction

Allogeneic stem cell transplantation (alloSCT) has been established as an important treatment modality for patients with hematological malignancies, aplastic anemia, and inborn errors of hematopoietic progenitor cells. Nevertheless, major lethal and non-lethal complications still prohibit a broader application of alloSCT.

Acute graft-versus-host disease (aGVHD) is a major cause of morbidity and mortality after alloSCT [1]. High dose systemic glucocorticosteroids (steroids) are currently recommended as first-line treatment for grade II-IV aGVHD resulting in overall complete responses (CR) in 40%-50% of patients [2, 3]. However, the likelihood to respond to treatment decreases with increasing severity of the disease. Patients with grade II aGVHD at diagnosis are significantly more likely to achieve a CR to initial treatment with high dose steroids as compared to patients with more advanced aGVHD [4, 5]. The prognosis of patients with aGVHD who fail to respond to high dose steroids is poor [2]. Currently there is no standard second-line treatment for steroid-refractory aGVHD (SR-aGVHD). Numerous strategies to treat SR-aGVHD have been reported, but results have been disappointing with varying response rates and long term overall survival (OS) of only 20–30% [2].

Tumor necrosis factor alpha (TNFα) is involved in the pathophysiology of aGVHD by activating antigen-presenting cells, recruiting effector cells and causing tissue damage [6]. Etanercept is a recombinant human tumor necrosis receptor fusion protein which binds TNFα with high affinity and as a consequence inhibits the biological activity of TNFα. Studies that have investigated the use of anti-TNFα as primary as well as secondary treatment in aGVHD have shown promising response rates. First-line combination with methylprednisolone yielded a CR rate of 69% [7] and second-line treatment resulted in an overall response rate (ORR) of 46% [8]. Second-line therapy combining etanercept with daclizumab showed an ORR of 67% [9]. Treatment with multi-agent combination therapy including etanercept has been reported to induce responses in 81% of patients [10]. Based on these data we implemented the use of etanercept as second-line treatment for SR-aGVHD. Here we report the results of a retrospective analysis of patients with SR-aGVHD treated with etanercept in our center.

## Patients and methods

We retrospectively studied 15 alloSCT patients treated with etanercept for SR-aGVHD at the Erasmus MC Cancer Institute between January 2009 and April 2013. The institutional review board approved the transplantation protocols, and all patients provided informed consent for stem cell transplantation and data collection. None of the transplant donors were form a vulnerable population and all donors or next of kin provided written informed consent that was freely given.

According to underlying disease and age, patients received either myeloablative, non-myeloblative or reduced-intensity conditioning regimens (conditioning intensities according to Bacigalupo [11]). Transplants were unmanipulated grafts form HLA-identical siblings (SIB) or at least 7/8 (A, B, C, DRB1) HLA-matched unrelated donors (MUD). Patients without a suitable SIB or MUD received an umbilical cord blood transplantation. In case of umbilical cord blood (UCB), a double UCB transplantation was performed using two at least 4/6 (A, B, DRB1) matched cord blood units.

### Acute GVHD: Prophylaxis, diagnosis, treatment and response criteria

Standard aGVHD prophylaxis for all allograft recipients included cyclosporin A (CsA) and mycophenolic acid (MPA) or its prodrug mycophenolate mofetil (MMF). In case of a 7/8 HLA matched MUD, anti-thymocyte globulin (ATG) was added to the conditioning regimen. Levels of CsA were measured by high performance liquid chromatography on whole blood samples and aimed at trough levels of 250–350 μg/L. Acute GVHD was graded according to the modified Glucksberg criteria [12], and the diagnosis of aGVHD was preferably confirmed by histology of involved tissues.

First-line treatment for grade II to IV aGVHD consisted of prednisolone 2mg/kg/day and CsA or MPA/MMF. Grade I aGVHD was treated with topical steroids.

Steroid refractoriness (SR) was defined as either progressive disease (PD) or mixed response (MR) after 7–14 days of first-line treatment, no response/stable disease (SD) after 10 days, or progression of initial partial response (PR) after 10 days of treatment.

### Etanercept

Once SR-aGVHD was established, second-line treatment with etanercept was initiated, in addition to first-line therapy. Etanercept (Enbrel, Pfizer) was administered subcutaneously twice weekly at a dose of 25 mg for an intended period of eight weeks.

### Antimicrobial prophylaxis

Prophylaxis for all patients included (val)acyclovir (herpes viruses) and cotrimoxazole (*Pneumocystis jirovecii*) until at least one year after transplantation or prolonged in case of GVHD. Patients with SR-aGVHD treated with etanercept received additional anti-aspergillus prophylaxis with voriconazole. Furthermore, since August 2012, patients with aGVHD of the lower gastrointestinal tract (irrespective of steroid responsiveness) with complaints of bloody diarrhea or large mucosal defects at endoscopy, received levofloxacin. Patients at risk for cytomegalovirus (CMV) and Epstein-Barr virus (EBV) reactivations were monitored by quantitative polymerase chain reaction (PCR) of plasma and treated preemptively according to local protocols.

### Outcome definitions

CR was defined as resolution of all signs and symptoms of aGVHD. PR was defined as improvement of 1 stage in 1 or more organs without progression in other organs. MR was defined as improvement in at least one organ with progression in at least one other organ. SD was defined as absence of improvement. PD was defined as progression in one organ with no improvement in any other organ.

### Statistical analysis

The primary endpoint of the study was the best overall response rate (CR and PR) after start of second-line treatment with etanercept. Secondary endpoints were OS and toxicity. OS was calculated using the actuarial method of Kaplan and Meier from the time of starting second-line treatment with etanercept until death. Causes of death were classified as treatment-related mortality (TRM) or relapse-related mortality.

## Results

### Patient characteristics

The characteristics of all fifteen patients treated with etanercept for SR-aGVHD are shown in Table 1. Median age was 54 years (range 22–65 years), eleven patients (73%) were male. One patient received a myeloablative conditioning (MA) regimen consisting of cyclophosphamide (2 x 60 mg/kg) and 2 x 6 Gray (Gy) total body irradiation (TBI). All other patients either received a reduced intensity conditioning (RIC) with cyclophosphamide (1 x 60 mg/kg), fludarabin (4 x 40 mg/m2) and TBI (2 x 2 Gy) or a non-myeloablative conditioning (NMA) with fludarabin (3 x 30 mg/m2) and TBI (1 x 2 Gy). Eleven patients received peripheral blood stem cells either from HLA identical sibling donors (n = 3) or from HLA matched unrelated donors (n = 8). Four patients received cord blood (CB) derived stem cells. One patient developed late-onset aGVHD after discontinuation of immunosuppressants, at day 393 post-transplant. All other patients were on GVHD prophylaxis at the onset of aGVHD. Acute GVHD developed at a median of 61 days after transplantation (range 23–393 days). All patients had grade III GVHD of the gut, the majority had involvement of a second organ. First-line treatment consisted of CsA and prednisolone 2/mg/kg/day in fourteen patients; one patient was treated with high dose steroids combined with MMF, because impaired renal function prohibited the use of calcineurin inhibitors.

**Table 1:** Patient characteristics.

*Case*	*Age*	*Sex*	*Diagnosis*	*Response*^*a*^	*Conditioning*^*b*^	*Donor*^*c*^
1	30	M	AML	CR	RIC	CB
2	51	M	NHL	SD	NMA	MUD
3	57	F	AML	CR	NMA	SIB
4	60	M	CLL	PR	NMA	MUD
5	53	F	ALL	CR	RIC	CB
6	59	M	MPD	SD	RIC	MUD
7	60	M	MDS	Untreated	NMA	MUD
8	39	F	AML	CR	MA	MUD
9	55	M	MPD	SD	RIC	MUD
10	65	M	AML	CR	RIC	MUD
11	54	M	NHL	CR	NMA	SIB
12	52	M	NHL	PR	RIC	CB
13	53	M	T-PLL	CR	NMA	MUD
14	22	M	HL	SD	NMA	SIB
15	61	F	MDS	Untreated	RIC	CB

^a^ Disease response status at transplantation

^b^ Conditioning regimens according to Bacigalupo

^c^ Stem cell source

Abbreviations

M = male, F = female

AML = acute myeloid leukemia, CLL = chronic lymphatic leukemia, MPD = myeloproliferative disease, MDS = myelodysplastic syndrome, NHL = non-Hodgkin lymphoma, FL = follicular lymphoma, T-PLL = T-cell prolymphocytic leukemia; HL = Hodgkin lymphoma

CR = complete remission, PR = partial remission, SD = stable disease

MA = myeloablative, NMA = non-myeloablative, RIC = reduced intensity conditioning

CB = cord blood donor, MUD = matched unrelated donor, SIB = sibling donor

### SR-aGVHD

#### Initiation of second-line treatment with etanercept

Second-line treatment with etanercept was initiated at a median of 13 days (range 5–36 days) after start of first-line treatment with high-dose steroids. Grades and organ involvement of aGvHD target organs at start of etanercept are shown in Table 2. High-dose prednisolone was maintained until achievement of objective GvHD response, with the exception of four cases in which severe infectious complications prompted earlier tapering. The median number of etanercept doses was 16 (range 1–23). Eight out of fifteen patients received at least all planned doses of etanercept, seven patients died while on therapy.

**Table 2:** aGVHD characteristics.

*Case*	*Prophylaxis*^*a*^	*GVHD onset*^*b*^	*GVHD stage*^*c*^			*GVHD overall grade*^*c*^	*First-line treatment*
			*skin*	*liver*	*gut*		
1	CsA	113	1	0	3	III	P/CsA
2	CsA/MMF	27	0	1	3	III	P/CsA
3	-	357	0	0	4	III	P/CsA
4	CsA/MMF	84	0	0	3	III	P/CsA
5	CsA	50	0	2	3	III	P/CsA
6	CsA	23	3	0	3	III	P/CsA
7	CsA/MMF	61	3	0	4	III	P/CsA
8	CsA/MMF	27	0	1	4	III	P/CsA
9	CsA/MMF	126	0	0	4	III	P/CsA
10	CsA/MMF	39	0	0	3	III	P/CsA
11	MMF	393	0	1	4	III	P/CsA
12	CsA	79	0	0	2	III	P/MMF
13	CsA/MMF	41	3	0	2	III	P/CsA
14	MMF/P	86	0	1	3	III	P/CsA
15	CsA	44	0	3	4	III	P/CsA

^a^ At time of GVHD onset

^b^ Days post-SCT

^c^ GvHD stage of aGvHD target organs at start etanercept

Abbreviations:aGVHD = acute graft-versus-host disease, CsA = cyclosporin A, MMF = mycophenolate mofetil, P = prednisolone

#### Response to second-line treatment with etanercept

Overall response rate was 53%, with 3 CR (20%) and 5 PR (33%) observed. Responses in GVHD were maintained in 2 out of 3 CR patients (66.7%). In the third CR patient etanercept had to be discontinued, because of EBV-reactivation and development of post-transplant lymphoproliferative disease (PTLD). He subsequently developed progressive GVHD, which proved refractory to further therapy. Response of GVHD was maintained in 1 of 5 PR patients (20%). This patient, however, succumbed to a second relapse of his original malignancy approximately 14 weeks after etanercept had been started. The other 4 PR patients lost their response and died of progressive SR-aGVHD. Results are shown in Table 3.

**Table 3:** Patient and GVHD outcomes.

*Case*	*Start ETN*^*a*^	*Best response*	*Outcome*	*FU*^*b*^	*COD*
1	13	PR	Dead	179	AML relapse
2	30	CR	Dead	141	GVHD
3	31	PD	Dead	41	GVHD
4	9	PR	Dead	47	GVHD
5	24	PR	Dead	267	Infection
6	11	PD	Dead	12	Infection
7	9	PD	Dead	6	Infection
8	20	PD	Dead	17	Infection
9	36	PR	Dead	89	GVHD
10	5	PD	Dead	48	GVHD
11	13	PR	Dead	87	GVHD
12	28	CR	Dead	109	Cardiac
13	19	PD	Dead	66	Infection
14	12	CR	Dead	89	Infection
15	5	PD	Dead	5	GVHD

^a^ Interval (days) between start of 1^st^ line treatment and introduction of etanercept

^b^ Follow-up (days) after starting etanercept

Abbreviations: GVHD = graft-versus-host-disease, ETN = etanercept, COD = cause of death, PR = partial response, PD = progressive disease, CR = complete remission, AML = acute myeloid leukemia.

### Complications

Clinically significant infectious complications (CTC-AE grade ≥3 or grade 2 requiring systemic treatment) occurred in 13 patients (87%), including bacterial sepsis in 9 patients (60%), viral infections in 8 patients (53%; including CMV and EBV reactivation, BK virus, rhinovirus, and coronavirus), and invasive pulmonary aspergillosis in 3 patients (20%). One patient experienced a seizure, which was attributed to CsA, rather than etanercept. Infectious complications are shown in Table 4.

**Table 4:** Infections.

*Case*	
1	None
2	EBV-PTLD
3	Sepsis *e*.*c*.*i*.; CMV reactivation
4	EBV reactivation
5	CMV reactivation; pulmonary aspergillosis; anal herpes simplex lesion
6	BK virus cystitis; *E*. *coli* sepsis
7	*Enterococcus faecium* sepsis
8	EBV reactivation
9	*Bacteroides fragilis* sepsis; CMV colitis
10	*E*. *coli* sepsis
11	*CNS* catheter-related sepsis; cellulitis
12	*Klebsiella pneumoniae* sepsis
13	Pulmonary aspergillosis/zygomycosis; rhinovirus; *Enterococcus faecium* sepsis
14	Disseminated aspergillosis; CMV reactivation; pulmonary infection (rhino/coronavirus and *Citrobacter freundii)*
15	None

Abbreviations:EBV = Epstein-Barr virus, PTLD = post-transplantation lymphoproliferative disorder, CMV = cytomegalovirus, CNS = coagulase negative streptococci, e.c.i. = e causa ignota

### Overall survival

**Fig 1:**
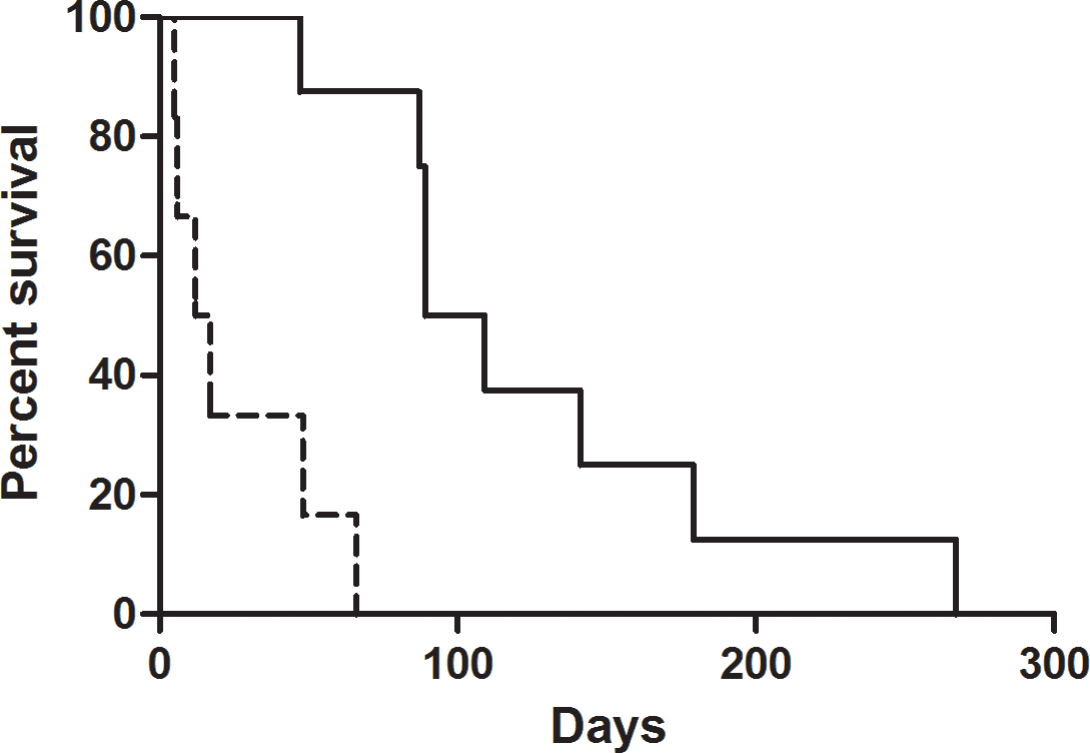
Survival by best response to treatment with etanercept. Survival in time for patients responding to treatment with etanercept (CR and PR; solid line) and patients not responding to treatment with etanercept (SD and PD; dotted line).

Median OS after initiation of second-line treatment with etanercept was 66 days (range 5–267) for the entire group. As shown in Fig 1, median OS was 99 days (range 47–267 days) for responders and 17 days (range 5–66 days) for non-responders (p<0.01). Eventually, all patients died.

Causes of death were progressive aGVHD in 7 patients (47%), infection in 6 patients (40%), relapsed acute myeloid leukemia in one patient (6.7%), and cardiac arrest in 1 patient (6.7%). In half of the patients who succumbed to an infection, ongoing GVHD was considered to be a contributing factor.

## Discussion

Acute GVHD is an important cause of morbidity and mortality following alloSCT. Steroid-refractory acute GVHD in particular is associated with a poor prognosis with a long term overall survival of only 20–30%. Studies evaluating second-line treatment with the anti-TFNα agent etanercept have shown promising results with overall response rates of 50–80% [8–10]. We performed a retrospective analysis in a small cohort of patients treated with etanercept for grade III SR-aGVHD of the gut. Second-line treatment with etanercept resulted in an encouraging overall response rate of 53%. Nevertheless, eventually all treated patients died, most importantly due to progression of GVHD and opportunistic infections.

Our results compare well to a recent prospective study published by Van Groningen et al. concerning 21 patients with SR-aGVHD treated with a combination of etanercept and inolimomab, an IL-2 receptor antibody [13]. This cohort was strikingly similar to ours with respect to age, aGvHD severity and proportion of patients with gut involvement. Despite an ORR of 48%, OS was only 10% at a median of 55 days, due to progressive GVHD, serious infections and relapse of underlying disease. The pattern of a promising initial response in about 50% of patients, followed by non-relapse mortality (NRM) in patients due to either progressive GVHD or the occurrence of serious infections, in particular, compares well to our findings. Wolff et al. prospectively studied the use of etanercept (16mg/m^2^) in combination with dacluzimab (1mg/kg), an IL-2 receptor antibody, in 21 patients with SR-aGVHD (9). This cohort was younger with a median age of 44 years, and included fewer patients with high-stage involvement of the gut. Although a promising ORR of 67% was observed, survival was disappointing as only 4 out of 21 patients survived.

Some studies, however, report better overall response and survival rates as compared to our cohort of patients. Kennedy et al. retrospectively studied the outcome of 16 patients, median age 42 years, with SR-aGVHD treated with a combination of ATG, tacrolimus and etanercept with or without MMF and reported an significantly higher ORR of 81% and OS of 50% [10]. In this analysis patients with less severe aGvHD were also included, with severity of gut GvHD ranging from stage 0 to 4. Xhaard et al. compared survival and infection rates in patients receiving MMF (56%), inolimomab (22%), or etanercept (23%) in addition to steroids and calcineurin inhibitors in SR-aGVHD [14]. The etanercept treated patients were younger than our cohort with a median age of 46 years (range 10–60 years). Most of them had high-stage gut involvement. Treatment response rate was 28% in etanercept treated patients. Two-year survival was 30% (95% CI: 22–41) and was not significantly different among the groups. Busca et al. reported on the use of etanercept in 13 SR-aGvHD patients [8]. This cohort compares well to ours with respect to age (median 52 yeara, range 26–70 years), but is more heterogeneous with respect to aGvHD severity and comprises fewer patients with stage 3 to 4 gut involvement. Overall response rate was 46% and 69% of patients were alive at a median follow-up of 429 days (range 71–1007 days).

In the publications reporting on etanercept as salvage therapy for SR-aGvHD, different approaches to tapering of high-dose steroids are being described, such as no tapering [13] to tapering starting at 14 days after start of etanercept [14]. Wolff et al. reported to start tapering from the onset of response, like we did.

The difference in ORR and survival rate as observed in these studies as compared to the present study might be explained by the type and grade of GVHD involved. Our study included a relatively high proportion of patients with severe stage III and IV aGVHD with bowel involvement, which is a well-known adverse prognostic factor for response to therapy [4]. In addition, the combination of different agents with etanercept in some studies and variable definitions of SR-aGVHD limit direct comparison of results. Moreover, interpretation of clinical response may be difficult, especially in case of GVHD of the gut.

Irrespective of response and survival rates, the rate of significant infectious complications is high in all reported studies. We report infectious complications in 81.3% of the patients. In 40% of patients, death was attributable to an infectious complication. The risk of opportunistic infections is known to be high in SR-aGVHD, due to the strong immunosuppressive regimen imposed on a frail, recovering post-transplant immune system. Anti-TNFα agents, in particular, are associated with a high incidence of opportunistic infections [15]. Moreover, the use of CB derived stem cells in four patients might have contributed, as transplantation of CB derived stem cells, in particular, is associated with delayed immune reconstitution [16]. Therefore, adequate monitoring and prophylaxis of infections is important.

In our study, invasive aspergillosis was the cause of death in 50% of patients that died of an infection. Susceptibility to aspergillus is known to be strongly increased by GVHD and immunosuppressive therapy [17]. Adequate prophylaxis by hospitalizing SR-aGVHD patients in high-efficiency particulate arrestance (HEPA)-filtered rooms if necessary and treatment with anti-fungal medication for the duration of immunosuppressive therapy is warranted. In the present study, all but one patient received antifungal prophylaxis at the time of initiation of etanercept. Thirteen patients received voriconazole and one patient was treated with amphotericin inhalations as elevated liver enzymes impeded the use of voriconazole. Despite prophylaxis, three patients developed invasive mould infections. The first patient developed an invasive aspergillosis despite amphotericin inhalations. The second patient proved to have a voriconazole-resistant aspergillus, and the third patient developed a double infection of voriconazole-resistant aspergillosis and zygomycosis.

Viral infections were observed in 9 out of 15 patients, with reactivation of CMV in 4 patients and EBV in 3 patients. In one patient a rapid rise in EBV viral load was accompanied by lymphadenopathy and a monoclonal B-cell population in the bone marrow. This EBV-lymphoproliferative disease was successfully treated with rituximab in combination with reduction of immunosuppression. CMV was monitored by PCR in patients at risk and treated pre-emptively with (val)ganciclovir. Nevertheless, one patient developed CMV-related colitis under pre-emptive treatment, probably related to the severe immunosuppressive state of our patients due to the GVHD itself and the immunosuppressive agents used. Episodes of septicemia were observed in 9 patients including four due to Gram-negative bacteria. All episodes of Gram-negative septicemia occurred either before the introduction of levofloxacin or were caused by less susceptible strains.

In conclusion, although second-line treatment of SR-aGVHD of the gut with etanercept was associated with a promising initial response rate, overall survival appeared very poor, mainly due to progression of GVHD and opportunistic infections. Alternative strategies to prevent and treat SR-aGVHD are urgently needed and prospective studies should be prioritized to improve the grim prognosis of SR-aGVHD.

## Abbreviations

aGVHD: acute graft-versus-host disease
alloSCT: allogeneic stem cell transplantation
ATG: anti-thymocyte globulin
CB: cord blood
CMV: cytomegalovirus
CR: complete response
CsA: cyclosporin A
EBV: Epstein-Barr virus
HLA: human leucocyte antigen
MA: myeloablative
MMF: mycophenolate mofetil
MPA: mycophenolic acid
MR: mixed response
MUD: matched unrelated donor
NMA: non-myeloablative
ORR: overall response rate
OS: overall survival
PCR: polymerase chain reaction
PD: progressive disease
PR: partial response
PTLD: post-transplantation lymphoproliferative disorder
RIC: reduced intensity conditioning
SB: stable disease
SIB: sibling
SR: steroid-refractory
TBI: total body irradiation
TNFα: tumor necrosis factor alpha
TRM: treatment-related mortality
UCB: umbilical cord blood
